# Autoregressive modeling to assess stride time pattern stability in individuals with Huntington’s disease

**DOI:** 10.1186/s12883-019-1545-6

**Published:** 2019-12-09

**Authors:** Helia Mahzoun Alzakerin, Yannis Halkiadakis, Kristin D. Morgan

**Affiliations:** Biomedical Engineering, School of Engineering, University of Connecticut, 260 Glenbrook Road, Storrs, CT 06269-3247 USA

**Keywords:** Huntington’s disease, Gait stability, Pattern analysis, Gait biomarkers

## Abstract

**Background:**

Huntington’s disease (HD) is a progressive, neurological disorder that results in both cognitive and physical impairments. These impairments affect an individual’s gait and, as the disease progresses, it significantly alters one’s stability. Previous research found that changes in stride time patterns can help delineate between healthy and pathological gait. Autoregressive (AR) modeling is a statistical technique that models the underlying temporal patterns in data. Here the AR models assessed differences in gait stride time pattern stability between the controls and individuals with HD. Differences in stride time pattern stability were determined based on the AR model coefficients and their placement on a stationarity triangle that provides a visual representation of how the patterns mean, variance and autocorrelation change with time. Thus, individuals who exhibit similar stride time pattern stability will reside in the same region of the stationarity triangle. It was hypothesized that individuals with HD would exhibit a more altered stride time pattern stability than the controls based on the AR model coefficients and their location in the stationarity triangle.

**Methods:**

Sixteen control and twenty individuals with HD performed a five-minute walking protocol. Time series’ were constructed from consecutive stride times extracted during the protocol and a second order AR model was fit to the stride time series data. A two-sample t-test was performed on the stride time pattern data to identify differences between the control and HD groups.

**Results:**

The individuals with HD exhibited significantly altered stride time pattern stability than the controls based on their AR model coefficients (AR1 *p* < 0.001; AR2 *p* < 0.001).

**Conclusions:**

The AR coefficients successfully delineated between the controls and individuals with HD. Individuals with HD resided closer to and within the oscillatory region of the stationarity triangle, which could be reflective of the oscillatory neuronal activity commonly observed in this population. The ability to quantitatively and visually detect differences in stride time behavior highlights the potential of this approach for identifying gait impairment in individuals with HD.

## Background

Huntington’s disease (HD) is a fatal neurodegenerative disorder for which there is presently no cure [1–4]. This neurodegenerative disorder leads to both cognitive and physical impairments due to the degeneration of nerve cells in the brain [3–5]. Gait, which is governed by the neuromuscular system, is altered due to these impairments that often manifest as reductions in gait speed, diminished step height and length and increased stride time variability [1, 5–7]. Unfortunately, as the disease progresses, these conditions intensify and significantly alter an individual’s gait stability [5, 6, 8]. Previous studies have related reduced stability in individuals with HD to an increased fall risk resulting from elevated stride time variability [8–10]. Nevertheless, a more direct relationship between temporal stride time patterns and gait stability has not, to our knowledge, been extensively explored. Therefore, this study sought to examine differences in stride time pattern stability between controls and individuals with HD using time series modeling.

Researchers traditionally employ metrics; such as, the standard deviation and coefficient of variation, to quantify gait variability in individuals with pathological conditions [7, 8, 11]. While these techniques successfully identify the magnitude of the variability, Hausdorff et al. (1997) found that there was also an underlying temporal pattern in stride time data that has not often been accounted for in this pathological population [7]. That study used Detrended Fluctuation Analysis (DFA) to show that stride time interval patterns were less correlated in individuals with HD as compared to controls groups [7]. DFA was able to identify such pattern trends by evaluating self-similarity among respective time series [12, 13]. Autoregressive (AR) modeling also assesses the strength of the self-similarity within a time series but additionally reveals a feature about dynamic stability [12, 13]. In AR modeling, a time series is dynamically stable if it is stationary [12, 13]. Stationary means that the time series’ mean, standard deviation and autocorrelation (self-similarity) do not change over time [12, 13]. Therefore, dynamically stable stride time patterns will exhibit constant stride times with no growth or decrease in stride time variability and a constant repeating pattern. The AR model coefficients capture these time series dynamics and are mapped to a stationarity triangle to visually denote how close their dynamics place them to critical transition states, which are the unstable or oscillatory states [12, 13]. The advantage of AR modeling is that the visual nature of the stationarity triangle allows individuals to not only denote if their dynamics are stable but how close their dynamics are to becoming unstable or oscillatory in nature. Exploiting this feature of AR modeling is the expressed aim of the current investigation.

AR modeling has successfully identified respiratory rates and fatigue levels from physiological signals, isolated differences in movement strategies from force data, and evaluated postural stability and identified fall risk from gait patterns [14–20]. The latter studies highlight how constructing a time series out of more traditional biomechanical measurements and characterizing its dynamics using the AR coefficients can help identify changes in movement pattern stability that are indicative of fall risk. Increased fall risk, which is also a concern for individuals with HD, is attributed to motor control deficits that are prevalent in this population [6, 21, 22]. These deficits cause abnormal involuntary movement (chorea) and an inability to regulate the timing of gait events; such as; stride times [6, 21–23]. Since AR modeling defines the current value as function as the previous values; changes in the relationship of current and past stride times; captured by the AR model coefficients, will provide insight into stride time regulation and motor control. Furthermore, the ability of AR coefficients to denote the transition to either oscillatory dynamics, which could present as abnormal involuntary movement, or unstable dynamics that could indicate elevated fall risk, indicate how the AR coefficients can detect motor control deficits in individuals with HD via stride time patterns.

The objective of this study was to use AR modeling to evaluate differences in stride time pattern between individuals with HD and controls during walking. Stride time pattern stability was deduced from the AR model coefficients for the individuals in the respective groups. It was hypothesized that individuals with HD would exhibit altered stride time pattern stability and reside in different locations within the stationarity triangle. The ability to delineate these differences would demonstrate how AR modeling can be used as a non-invasive diagnostic tool to help monitor disease progression.

## Methods

The data analyzed in this study was collected by researchers at the Massachusetts General Hospital (MGH) where they recruited participants from the Neurology Outpatient Clinic to perform a walking protocol [7]. Every participant provided written consent to participate in the study in accordance with the MGH institutional review board [7]. The researchers provided access to the deidentified study data on an online database (https://www.physionet.org/physiobank/database/gaitndd/). The data from that database was used for this analysis [7].

### Instrumented gait analysis

Sixteen control (height 1.8 ± 0.1 m; mass 66.8 ± 11.1 kg; age 39.3 ± 18.5 yrs.; speed 1.4 ± 0.2 m/s) and twenty individuals with HD (height 1.8 ± 0.1 m; mass 72.1 ± 17.0 kg; age 47.7 ± 12.6 yrs.; speed 1.1 ± 0.3 m/s) performed a walking .protocol. The participants were instructed to walk at a self-selected speed along a hallway that was 77 m in length. Once at the end of the hallway, participants would turn around and walk back towards the other end. Each participant walked up and down the hallway for 5 min. The control participants were free of any neurological and physical conditions that would prevent them from participating or alter their ability to perform the walking protocol. Neurologist from the clinic assessed the individuals with HD. The severity of HD was evaluated using the total functional capacity (TFC) score from the Unified Huntington’s Disease Rating Scale. The TFC score ranges from 0 to 13 where a 0 indicates individuals with the most severe impairments, and a 13 indicates individuals with little to no impairment [7, 24].

Force sensitive resistors were embedded in the individual’s shoes. These resistors measured the force produced during each step and was collected at 300 Hz. These resistors allowed the researchers to record gait data. From this gait data, the researchers were able to identify initial contact and toe off and these measurements allowed for the identification and extraction of stride interval data.

### Time series stability analysis

**Fig. 1:**
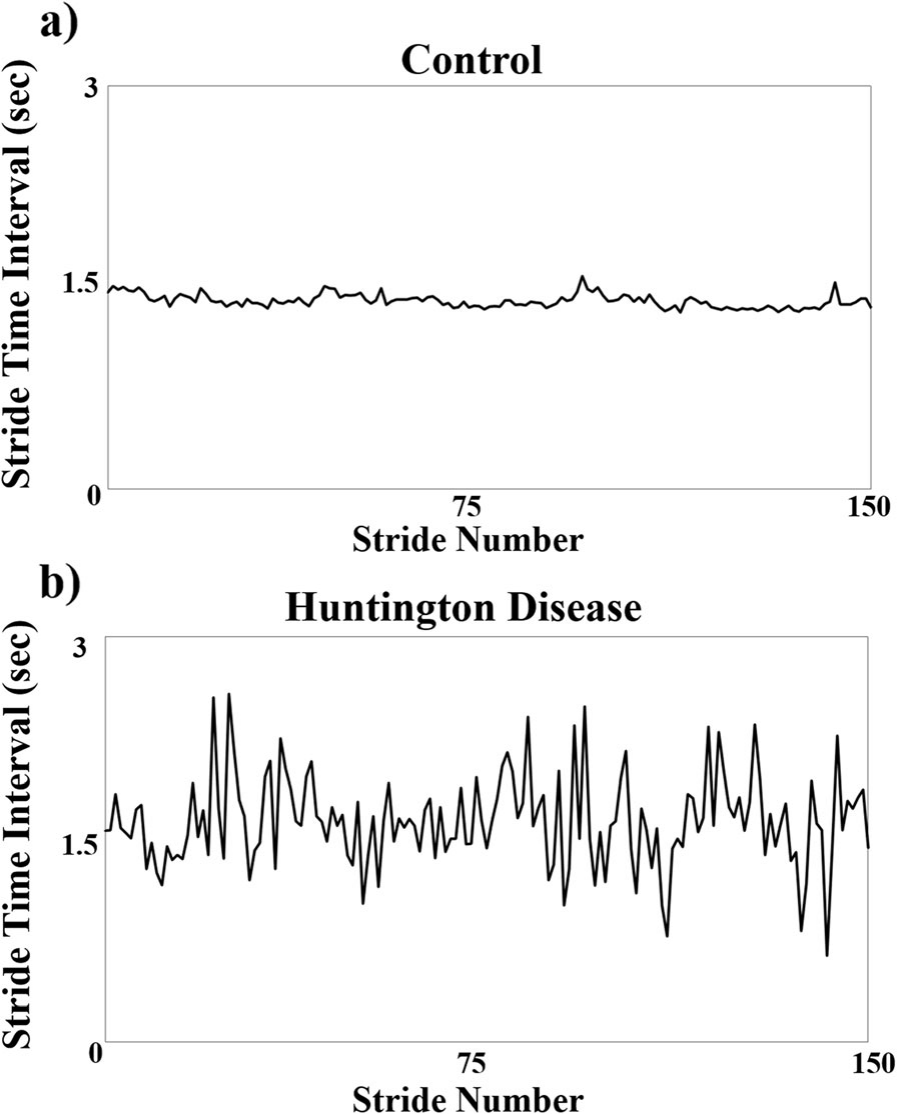
Walking stride time interval patterns for an individual in the (**a**) control group and (**b**) an individual in the HD group. Each figure represents the constructed stride time interval time series, which plotted the strides as a function of stride time

The consecutive stride times from the five-minute walking protocol were combined to form a time series for each individual (Fig. 1). The first step in the AR model analysis involved subtracting a linear trend from the stride time interval time series. Next, a second order AR model (AR(2)) was fit to the detrended time series (Eq. 1).

1 $$ {y}_t=\delta +{\varnothing}_1{y}_{t-1}+{\varnothing}_2{y}_{t-2}+{\varepsilon}_t $$

Here *y*_*t*_ represents the current value, *y*_*t* − 1_ and *y*_*t* − 2_ are the values of the two previous time steps, ∅_1_ and ∅_2_ are the AR1 and AR2 coefficients, respectively, for the two previous time steps, *δ* is a model constant and *ε*_*t*_ is white noise [12, 13]. The order of the AR model was determined from the results of the autocorrelation function (ACF) and partial autocorrelation (PACF) function plots [12, 13]. The AR model coefficients indicate how strongly correlated the current value is with the previous two values and shows how well the previous states predict the current model state. Furthermore, the AR coefficients indicate if and how quickly the time series is transitioning into a nonstationary process. A time series is stationary if the mean, variance and autocorrelation behavior remain constant over time [12, 13]. Since the time series are constructed from stride times, the AR model coefficients represent the gait dynamics. The two coefficients obtained from the AR(2) model, AR1 and AR2, were used as the x-axis and y-axis coordinates, respectively, of the point plotted on the stationarity triangle.

The stationarity triangle is used to evaluate the behavior and stability of a time series. Any point that lies outside of the stationarity triangle indicates that the time series produces unstable dynamics [12, 13]. For those points that lie within the stationarity triangle, there are two possible stable dynamic outcomes. Those points that lie within the semicircle inside of the stationarity triangle produce an oscillatory response that generates stable harmonic motion [12, 13]. Points that lie external to the semicircle indicate a non-oscillatory response and represent stable damped motion that possesses two embedded time constants [12, 13]. The distance from the points to the centroid of the stationarity triangle at (0, − 1/3), which resides in the oscillatory region, is used to indicate the stability and dynamic behavior of the time series. Distances closer to the centroid indicate that the time series is stable and exhibits more oscillatory behavior and the distances further away from the centroid indicate that the time series is stable but exhibits non-oscillatory behavior. The distance metric is unitless because the AR(2) coefficients, from which the distance is calculated, are dimensionless. Ellipses are drawn around the controls and HD individuals, respectively, to further delineate the different dynamic responses between the two groups. These ellipses encircled 95% of the individuals in their respective groups. All of the aforementioned analyses were conducted using a custom MATLAB code (MATLAB R2018a, The MathWorks, Inc., Natick Massachusetts, USA).

### Statistical analysis

A two-sample t-test was conducted to test the hypothesis that there were no differences in the means in mean age, height, mass, gait speed, stride time, and stability metrics - AR1, AR2 and AR stability distance – between the controls and individuals with HD (α = 0.05). The performance of the metrics was based on model accuracy, specificity and sensitivity. All statistical analyses were conducted in Minitab (Minitab 16, Minitab Inc., State College, Pennsylvania, USA.).

## Results

No differences in age, height, and mass were found between the controls and individuals with HD (Table 1). However, the individuals with HD walked at 1.1 ± 0.3 m/s which was significantly slower (*p* = 0.04) than the controls who walked at 1.4 ± 0.2 m/s (Table 1). Consequently, the individuals with HD had significantly (*p* = 0.04) longer stride times (1.2 ± 0.2 s) than the controls (1.1 ± 0.1 s) (Table 2). This indicated that while the individuals with HD walked at a slower speed, they exhibited greater stride time variability than the controls as noted in the standard deviation values.

**Table 1:** Comparison of participant demographics. (Mean ± Standard Deviation)

Variable	Control Group	HD Group	*P*-value
Age (years)	39.3 ± 18.5	47.7 ± 12.6	0.17
Height (m)	1.8 ± 0.1	1.8 ± 0.1	0.94
Mass (kg)	66.8 ± 11.1	72.1 ± 17.0	0.30
Speed (m/s)	1.4 ± 0.2	1.1 ± 0.3	0.04*
Total Functional Capacity		6.8 ± 3.9	
Gender (Female:Male)	14:2	14:6	

* Denotes that the means between the two groups were significantly different (α = 0.05)

**Table 2:** Comparison of stride time intervals, autoregressive modeling coefficients and distance metrics. (Mean ± Standard Deviation)

Variable	Control Group	HD Group	*P*-value
Stride Time (s)	1.1 ± 0.1	1.2 ± 0.2	0.04*
AR 1 Coefficient	0.4 ± 0.1	0.1 ± 0.2	< 0.001*
AR 2 Coefficient	0.1 ± 0.1	0.0 ± 0.1	< 0.001*
AR Distance	0.6 ± 0.1	0.4 ± 0.1	< 0.001*

* Denotes that the means between the two groups were significantly different (α = 0.05)

**Fig. 2:**
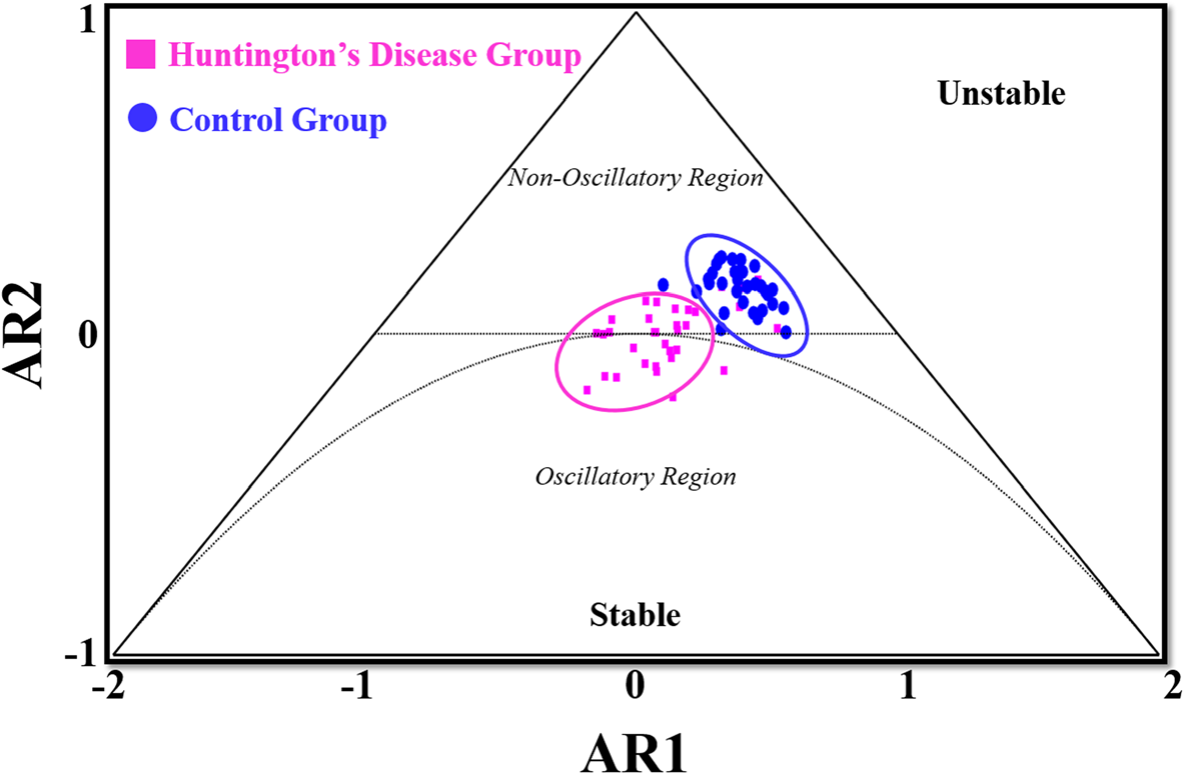
Comparison of stride time patterns for controls and individuals with HD on the AR(2) stationarity triangle. The blue circles represent the control individuals and pink squares represent the individuals with HD. The semicircle denotes the edges of the oscillatory region. The blue and pink ellipses encompass 95% of the individuals in the control group and the Huntington’s group, respectively

The individuals with HD exhibited significantly different stride time pattern stability than the controls (*p* < 0.001) based on the AR1, AR2 and the AR distance metrics (Table 2). The individuals with HD exhibited more oscillatory stride time patterns than the controls, who exhibited non-oscillatory behavior, as determined by their location in the stationarity triangle (Fig. 2). The controls were significantly further away from the centroid of the triangle in both the AR1 or x-direction and AR2 or y-direction than the individuals with HD (*p* < 0.001; *p* < 0.001). The individuals with HD also exhibited more variability in the AR1 direction than the controls. Overall, the control individuals were located 0.6 ± 0.1 away from the centroid of the triangle and the individuals with HD were 0.4 ± 0.1 away from the centroid (*p* < 0.001) (Table 2). Eighty percent of the individuals who exhibited reduced cognitive and physical impairment resided outside of the oscillatory region while 60% of the individuals who exhibited higher cognitive and physical impairment resided inside of the oscillatory region.

## Discussion

The objective of the study was to evaluate stride time pattern stability in controls and individuals with HD using AR modeling. The results supported the hypothesis as the individuals with HD exhibited significantly altered stride time pattern stability compared to the controls based on their location in the stationarity triangle. The individuals with HD resided closer to and inside of the semicircle of the stationarity triangle indicating that they exhibited more oscillatory stride time dynamics than the controls, who resided in the non-oscillatory region. This oscillatory behavior observed in the individuals with HD is consistent with previous research where they described similar oscillatory behavior in the same population as increased fluctuations and variability [7]. These changes in gait behavior are characteristic of individuals with HD who exhibit impaired and jerky movements [1, 25]. The AR modeling technique was able to both quantify and visually delineate differences in stride time stability between the controls and individuals with HD and further determined that those individuals exhibited oscillatory stride time dynamics.

HD affects the basal ganglia in the brain which is responsible for regulating the control of voluntary movements such as walking [25–27]. In the basal ganglia, the presence of HD is denoted by increased oscillatory neuronal activity, which here was observed as oscillatory stride time pattern stability [25, 27]. The oscillatory motion represents an inability to voluntary control one’s movements and indicates the presence of chorea or dystonia in the individual [28, 29]. Chorea and dystonia indicate the degeneration of nerve cells in the brain and as the disease progresses it is possible to observe an increase in oscillatory movement which more than likely would be associated with a lower TFC value [7, 24]. Given that individuals with HD who exhibit oscillatory gait patterns clustered near the oscillatory region of the stationarity triangle, this finding demonstrates that this diagram could be used track the progression of neurodegeneration through the 5 stages of HD. Furthermore, the fact that the majority of individuals with HD who are identified as having low physical and cognitive impairment based on the TFC scale resided outside of the oscillatory region and outside of the control region indicates that this tool is able to identify changes in motor control. Since the AR modeling coefficients were able to accurately identify and visually delineate differences in gait dynamics between the two groups supports this metric as a more quantitative and evaluative tool to monitor disease progression as it relates to gait disturbance.

Chorea and dystonia, which are responsible for involuntary, abnormal movements, have a debilitating effect on individuals with HD motor control and contributes to their difficulty in regulating the timing of gait events [6, 21]. Thus, analyzing stride time dynamics provides a non-invasive way to evaluate the effect of chorea and dystonia on motor control. AR modeling was an appropriate technique because its coefficients indicate the strength of the relationship between the current and previous stride times. The smaller AR coefficients magnitude in the HD group indicated reduced consistency in the temporal stride time patterns which is in line with the poor regulation of the timing of gait events that plagues this population [21].

The study is not without its limitations. First, individuals with HD walked significantly slower than the controls and had significantly slower stride times, which could be assumed to influence their stride time stability. While decreased gait speed is associated with individuals with HD, it did not influence the AR model results. The AR model assessed the stride time pattern, not the mean stride time. Furthermore, the trend was removed from the time series prior to the AR modeling analysis, therefore, the AR model only evaluated how the stride times changed from step to step. Second, a few of the individuals with HD resided in the same region of the stationarity triangles as the controls. However, these individuals were found to have higher TFC scores, which indicated reduced or no cognitive and physical impairments. Third, a secondary analysis that compared those with amyotrophic lateral sclerosis (ALS) and Parkinson’s to controls did not reveal the same level of discrimination as the individuals with HD did when also using AR modeling. The alternate dispersion patterns of the ALS and Parkinson’s groups may indicate how motor control is altered differently amongst individuals in these groups. However, AR modeling is an established statistical technique that has been utilized to identify alternate movement patterns and motor controls in different populations [12–20].

The results of the study established how AR modeling can be used to delineate differences in stride time pattern stability between controls and those with HD. Individuals with HD exhibited altered stride time stability compared to the controls. Their altered stride time pattern was oscillatory in nature and was reflective of the oscillatory brain activity previously measured in individuals in this population. The sensitivity of this metric to detecting these changes in gait patterns suggests it could be very beneficial in helping to monitor individuals with different pathological conditions. Future work should investigate the AR model’s ability to identify differences in gait patterns among groups with different pathological conditions.

## Conclusions

The purpose of this study was to evaluate stride time pattern stability in controls and individuals with HD via AR modeling. AR modeling was able to both quantitively and visually delineate differences in gait patterns between the controls and HD. Specifically, the AR modeling was able to detect oscillatory movement patterns in those with HD. These findings indicate how AR coefficients could be used to help monitor disease progression.

## Data Availability

The deidentified datasets generated and/or analyzed during the current study are available in the PhysioNet Database repository, https://www.physionet.org/physiobank/database/gaitndd/.

## Abbreviations

AR: Autoregressive
HD: Huntington’s disease
